# Annotating long intergenic non-coding RNAs under artificial selection during chicken domestication

**DOI:** 10.1186/s12862-017-1036-6

**Published:** 2017-08-15

**Authors:** Yun-Mei Wang, Hai-Bo Xu, Ming-Shan Wang, Newton Otieno Otecko, Ling-Qun Ye, Dong-Dong Wu, Ya-Ping Zhang

**Affiliations:** 1 State Key Laboratory of Genetic Resources and Evolution, Kunming Institute of Zoology, Chinese Academy of Sciences, Kunming, 650223 China; 2 University of Chinese Academy of Sciences, Beijing, 100049 China

**Keywords:** Artificial selection, Domestication, Long intergenic non-coding RNA, Chicken, Population genome, Transcriptome

## Abstract

**Background:**

Numerous biological functions of long intergenic non-coding RNAs (lincRNAs) have been identified. However, the contribution of lincRNAs to the domestication process has remained elusive. Following domestication from their wild ancestors, animals display substantial changes in many phenotypic traits. Therefore, it is possible that diverse molecular drivers play important roles in this process.

**Results:**

We analyzed 821 transcriptomes in this study and annotated 4754 lincRNA genes in the chicken genome. Our population genomic analysis indicates that 419 lincRNAs potentially evolved during artificial selection related to the domestication of chicken, while a comparative transcriptomic analysis identified 68 lincRNAs that were differentially expressed under different conditions. We also found 47 lincRNAs linked to special phenotypes.

**Conclusions:**

Our study provides a comprehensive view of the genome-wide landscape of lincRNAs in chicken. This will promote a better understanding of the roles of lincRNAs in domestication, and the genetic mechanisms associated with the artificial selection of domestic animals.

**Electronic supplementary material:**

The online version of this article (doi:10.1186/s12862-017-1036-6) contains supplementary material, which is available to authorized users.

## Background

The domestication of wild animals has dramatically impacted human life, allowing a shift from hunter-gatherer to farming societies, and promoted the rise of human civilization. Driven by artificial selection, domestic animals generally display many phenotypic changes in behavior, morphology, and physiology compared to their wild ancestors. Consequently, there is a very high phenotypic diversity among chicken breeds than any other bird species. These phenotypic variations are valuable resources for studying the evolution of complex genetic traits. However, previous studies on the genetic mechanisms underlying the evolution of complex traits have mainly focused on protein-coding genes [1–4]. A major reason is that the functional annotation of long non-coding RNAs (lncRNAs) is largely missing.

LincRNAs comprise a heterogeneous subset of RNAs that are longer than 200 nucleotides (nt) and are transcribed from intergenic regions without protein-coding potential. An increasing number of investigations have shown that many lincRNAs are not just transcriptional ‘noise’, but execute important functions in numerous biological processes including: transcriptional regulation [5–8], cell cycle and apoptosis [9, 10], as well as pluripotency and differentiation control [11, 12]. While the sequences and expression levels of most lincRNAs evolve rapidly, they are tissue-specific [13–15], but some show clear evolutionary conservation with strong purifying selection in exonic sequences or promoter regions [16]. LincRNAs have high relative abundance in genome, for example, lincRNAs accounted for more than half of chicken lncRNAs identified by Kuo et al. [17], but sparse information on their functional background. Thus, extensive research is required to fully define and integrate lncRNAs into genome biology.

In addition to its tremendous economic importance as a food source, chicken serves as a model organism, particularly to evolutionary biologists interested in investigating artificial selection of complex traits [18]. Similar to other domestic animals, studies of chicken domestication have primarily focused on protein-coding genes [1–4], and little attention has so far been paid to non-coding regions. Hence, the potential of lncRNAs to advance our understanding of the genetic mechanisms underlying diverse chicken phenotypes as well as other complex traits remains largely untapped.

In this study, we leveraged large-scale RNA-sequencing data (more than 800 transcriptomes) to unearth thousands of lincRNAs in chicken, which we then utilized to investigate their impact on domestication. We annotated the functions of these lincRNAs and found 68 that were differentially expressed under different conditions. We also factored into our analyses significant trait-correlated single-nucleotide polymorphisms (SNPs) from previous genome-wide association studies (GWAS) of chickens [19–38]. This analysis enabled us to uncover 47 lincRNA genes that have significant SNPs associated with special phenotypes. Based on population genetics analyses of SNPs in red junglefowls (RJFs) and domestic chicken genomes, we identified 419 lincRNAs that exhibit significant genetic differentiation between the two populations. Our study provides important insights towards a better utility of lincRNAs in studying domestication and artificial selection events.

## Results

### Constructing the lincRNA gene repertoire in the chicken genome based on 821 transcriptomes

**Fig. 1:**
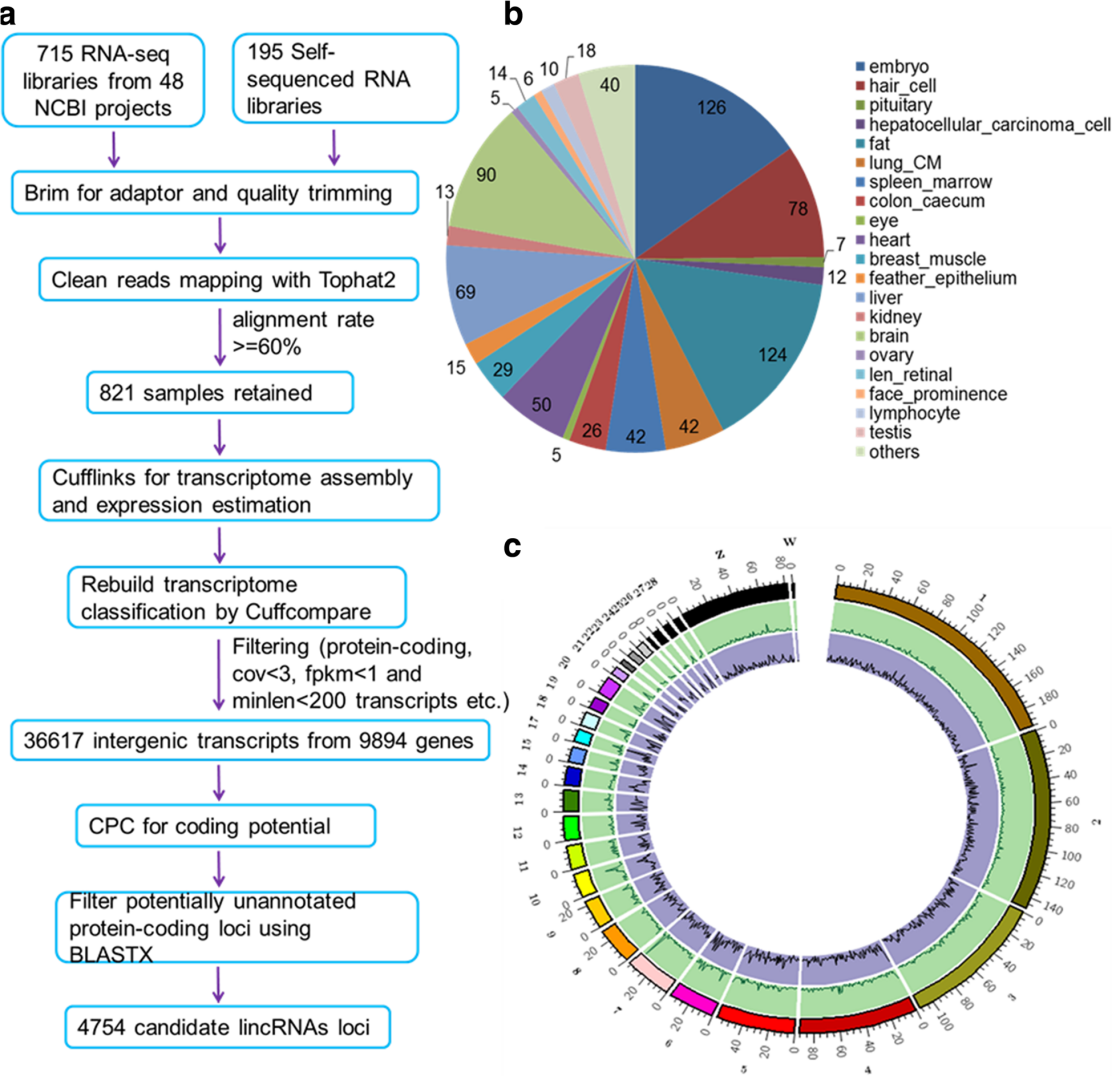
Identification of lincRNA genes in the chicken genome. **a**: Pipeline used to identify lincRNAs. **b**: Different groups of transcriptomes used in this study. **c**: Genome-wide landscape of lincRNA in the chicken genome

In order to capture the spectrum of chicken transcriptional diversity, we curated 715 RNA-seq libraries from 48 public datasets available in the National Center for Biotechnology Information (NCBI) database. We added another 195 RNA-seq libraries from our own projects. We excluded 89 libraries with mapping rates lower than 60%, and kept 821 for further analysis (Fig. 1a and Additional file 1). The reserved libraries comprise 21 cohorts that are mainly differentiated based on organ type and developmental stage (Fig. 1b, Additional files 2 and 3).

Following the procedure described in Fig. 1a and Methods, we credibly identified a total of 8134 transcripts from 4754 candidate lincRNA loci, encompassing almost all chromosomes [39] (Fig. 1c). Additionally, we identified 2942 novel putative lincRNAs not previously reported in two lncRNA databases, a domestic-animal long noncoding RNA database (ALDB) [40] and NONCODE (Additional file 4: Figure S1A).

Based on maximum expression levels across the 821 samples, lincRNAs exhibited lower expression levels than protein-coding genes, with an approximately 13 times lower median Fragments Per Kilobase of exon model per Million mapped fragments (FPKM) value (Additional file 4: Figure S1B). Meanwhile, our results show that the lengths of chicken lincRNA transcripts vary between approximately 200 base pairs (bp) and 1.5 kilobases (kb), with a median of about 1 kb, while protein-coding RNAs have a median length of approximately 3 kb (Additional file 4: Figure S1C and Additional file 5). Averagely, about 2.6 exons are present in lincRNA transcripts, far less than in protein-coding RNAs that have approximately 11 exons (Additional file 4: Figure S1D and Additional file 5). These findings indicate that chicken lincRNA transcripts are shorter and have fewer exons than protein-coding transcripts. A similar trend has been cited in human and mouse [41, 42] and further verified by our reanalysis (Additional file 5 and Additional file 6: Figure S2).

### Functional annotation of lincRNA genes in chicken

**Fig. 2:**
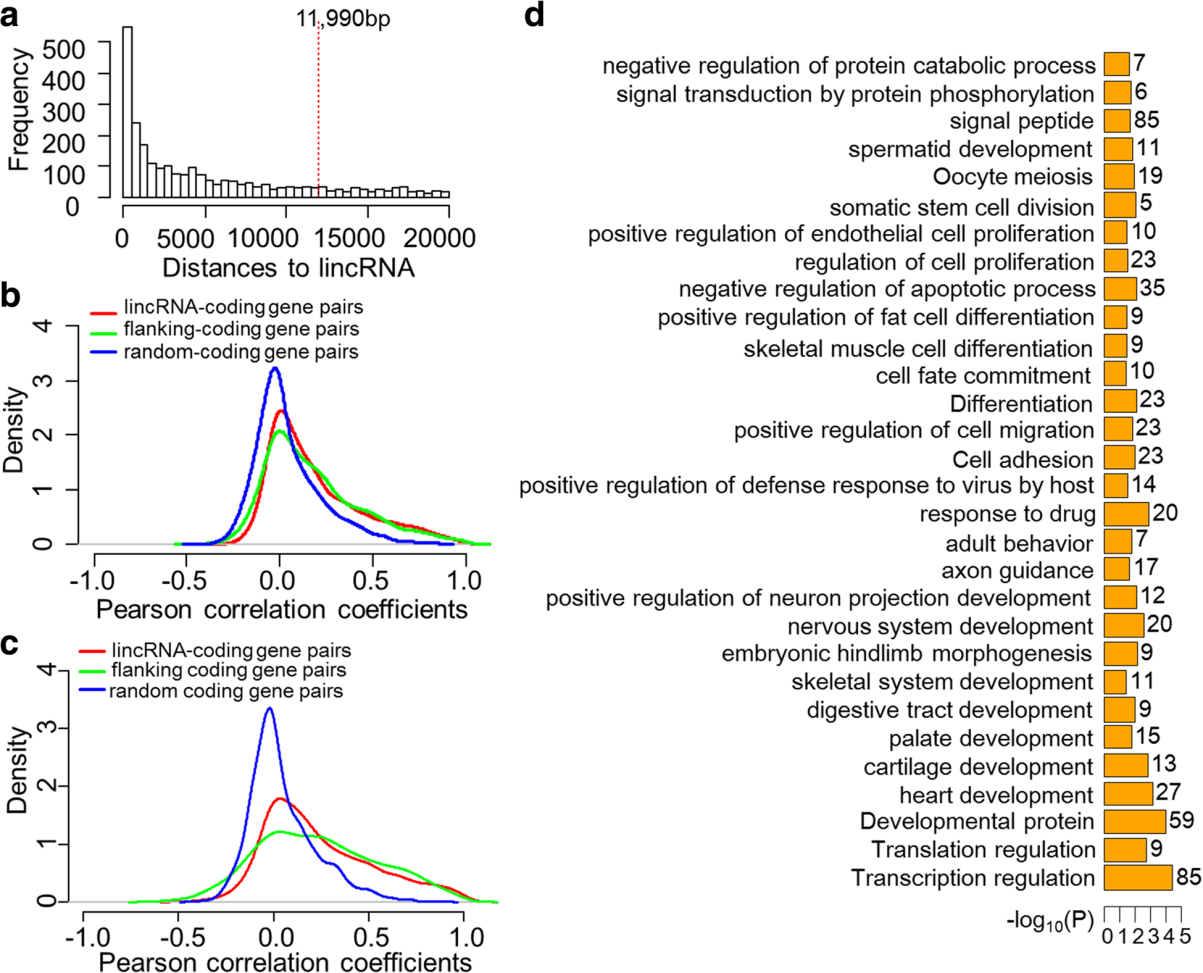
Functional annotation of lincRNAs based on adjacent protein-coding genes. **a**: Overview of distances from lincRNAs to their proximal protein-coding genes within 20 kb. Red dashed line at about 12 kb shows the median distance for all flanking protein-coding genes. **b**: Density of expression correlations between different gene groups by all samples. **c**: Density of expression correlations between different gene groups by 162 high quality samples. **d**: Significant categories enriched among protein-coding genes adjacent to lincRNAs within 20 kb

To investigate the potential functions of lincRNAs, we annotated transcripts based on flanking protein-coding genes. It has been reported that numerous lincRNAs influence the expression of adjacent protein-coding genes [43]. Our results indicate that the median distance between lincRNAs and their proximal protein-coding genes is approximately 12 kb. About 59% of flanking protein-coding genes is within 20 kb (Fig. 2a). A Pearson correlation coefficient (PCC) of FPKM across all samples indicates that expression correlations between lincRNAs and their proximal protein-coding genes are stronger than protein-coding gene pairs selected randomly (both *t*-test and Wilcoxon test *p*-value <2.2e-16), but similar to neighboring protein-coding gene pairs (*t-*test *p*-value = 0.07 and Wilcoxon test *p*-value = 0.09, Fig. 2b). This pattern is synonymous with that seen in other vertebrates including humans and zebrafish [42, 44, 45]. A more evident result was realized when only 162 high quality samples (RNA Integrity Number, RIN, ≥8.0, and mapping rates ≥80%) were analyzed (Fig. 2c).

The strong correlations between expression of lincRNAs and proximal protein-coding genes illustrate the presence of possible functional correlations as noted in previous studies [46, 47]. Therefore, we performed gene ontology (GO) enrichment using the database for annotation, visualization and integrated discovery (DAVID v6.7) for 1797 protein-coding genes located within 20 kb proximity to lincRNAs. This analysis showed enrichment for categories encompassing the development of the nervous system, palate, and heart, as well as the differentiation of brown fat cells and osteoblasts, the regulation of transcription and translation, apoptosis, proliferation, cell motility, and signal transduction (Fig. 2d). We also applied DAVID annotation for each tissue type by using parts of 1797 protein-coding genes that are within 20 kb proximity to lincRNAs and expressed in the tissue type (Methods). We observed enrichment for most of the categories stated before (Additional files 7 and 8). These results indicate that the lincRNAs identified in this study are likely involved in critical biological processes including: development, differentiation, cell proliferation, cell death, signaling, and transcriptional activity.

### Tissue-specific analyses of lincRNAs

**Fig. 3:**
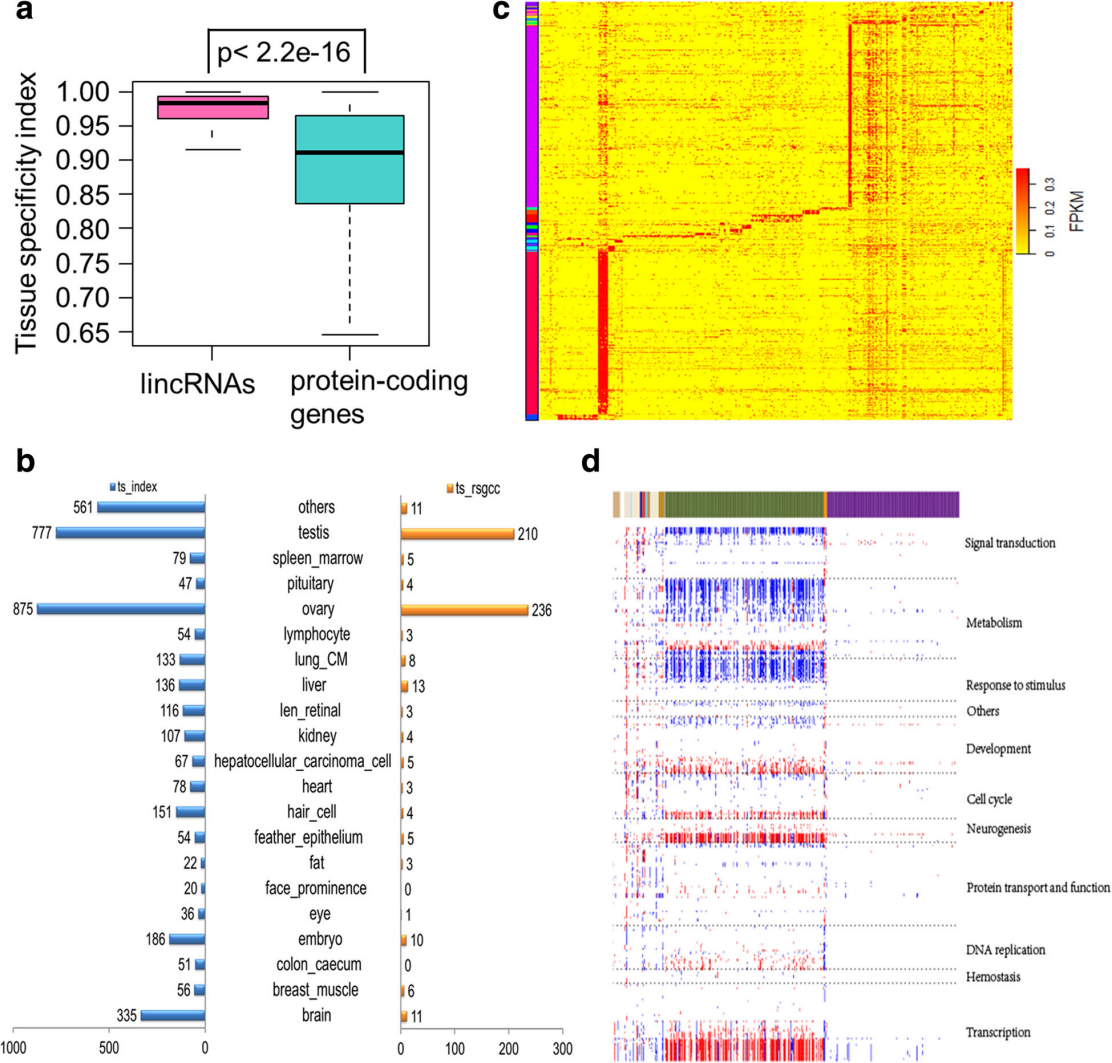
Tissue specificity of lincRNAs. **a**: TSI of lincRNAs and protein-coding genes. **b**: Number of tissue-specific lincRNAs identified from the TSI method (*blue*) and ‘rsgcc’ package (*orange*), respectively. **c**: Expression heatmap of tissue-specific lincRNAs identified using ‘rsgcc’. Columns represent samples while rows represent lincRNAs. **d**: Expression-based correlation matrix of 534 tissue-specific lincRNAs from ‘rsgcc’ (*column*) and 340 GSEA GO gene sets (*row*). Blue denotes a negative correlation, red a positive association, and white shows no significant relationship. These GO sets were divided into 11 clusters (*right*) based on their functional similarity and correlation

A number of previous studies have proposed that lncRNAs exhibit high tissue specificity [13, 42, 45], implying that many of these transcripts might function in a tissue specific fashion. Thus, to further assess the potential functions of lincRNAs, we computed the tissue specificity index (TSI) for expression patterns of lincRNAs and protein-coding genes [48]. TSI values ranged from 0 in housekeeping genes to 1 in tissue-specific genes. As expected, the results show that lincRNAs are more tissue-specific than protein-coding genes (Fig. 3a). Moreover, protein-coding genes flanking tissue-specific lincRNAs exhibit higher levels of tissue specificity than other protein-coding genes (Additional file 9: Figure S3A–C).

Because our data originated from different tissues with divergent sampling backgrounds, we applied weighted gene co-expression network analysis (WGCNA) in R software [49] as well as sample similarity to categorize the 821 samples into 21 tissue-related cohorts depending on gene expression values (Fig. 1b, Additional file 3 and Methods).

We then employed two strategies to retrieve tissue-specific lincRNAs. First, a lincRNA with TSI greater than or equal to 0.95 and whose highest FPKM value occurred in one tissue-related group was considered to be the tissue-related group’s specific lincRNA (designated as tissue-specific lincRNA) [50]. This analysis resulted in the identification of 3380 tissue-specific lincRNAs (Fig. 3b, Additional file 9: Figure S3D and Additional file 10). Secondly, 534 further tissue-specific lincRNAs were obtained by utilizing the ‘rsgcc’ package in R software [51] (Methods), and were all included in the TSI set (Fig. 3b–c and Additional file 10).

Tissue-specific lincRNAs can augment tissue-specific gene signatures [52]. Thus, we estimated Pearson correlations for expression levels between each specific lincRNA and all protein-coding genes across all samples in associated tissue cohorts by paired comparison. Protein-coding genes with significant *p*-values (< 0.05) and ranked PCCs were used for gene set enrichment analysis (GSEA) [53]. An association matrix [16, 43, 52] was built between 534 tissue-specific lincRNA genes and 340 significant GO gene sets (false discovery rate, FDR < 0.25). The GO sets were roughly clustered into 11 groups based on functional similarity and correlations. These groups included signal transduction, metabolism, response to stimuli, and cell cycle (Fig. 3d).

### Differentially expressed lincRNAs under different conditions

**Fig. 4:**
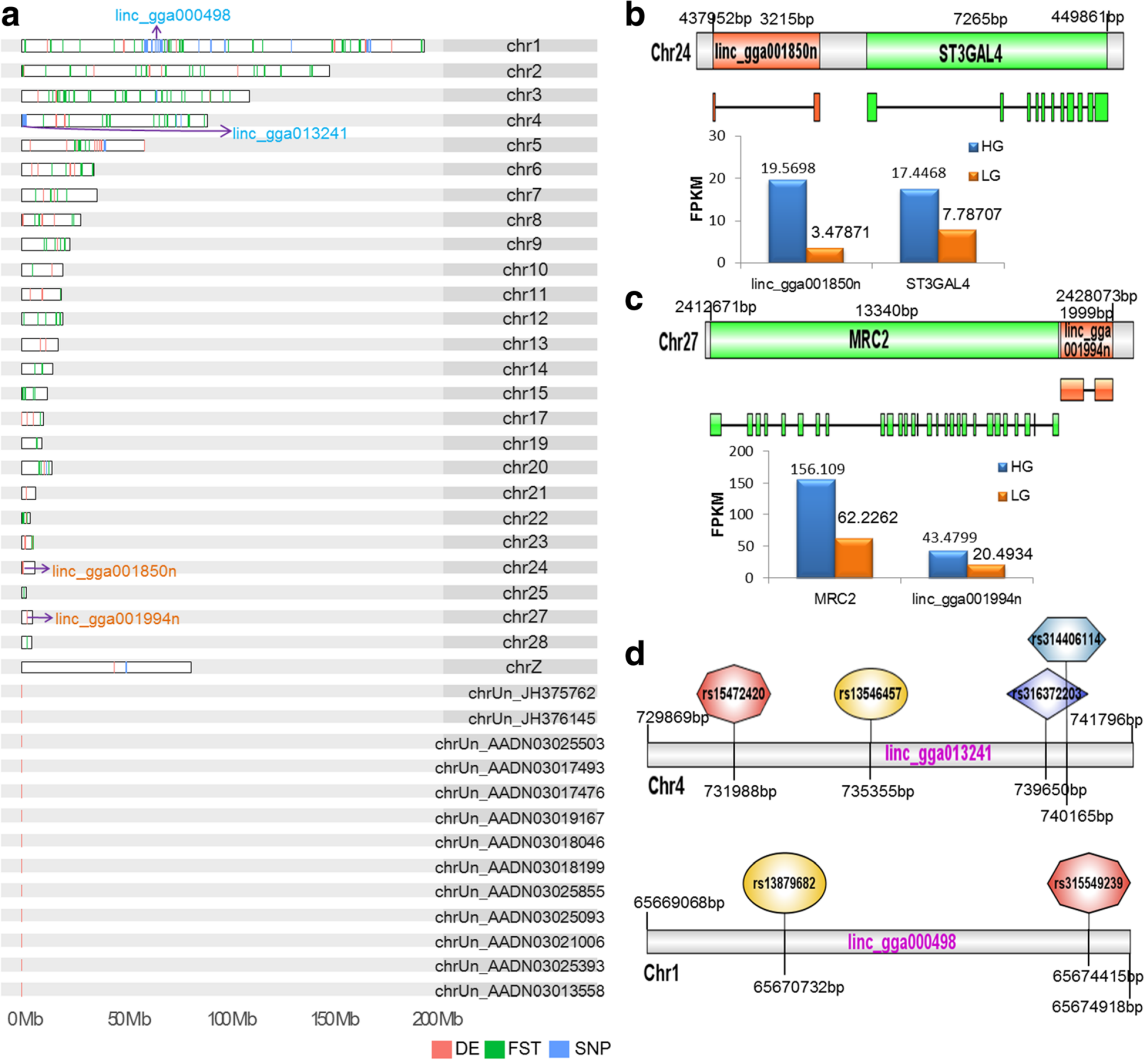
Chromosomal features of potentially functional lincRNAs. **a**: Chromosome landscape of 68 differentially expressed lincRNAs (*red*), 47 lincRNAs containing significant phenotype associated SNPs (*blue*), and 216 lincRNAs exhibiting high level population differentiation between domestic chicken and RJF (top 5% *F*_ST_ value) (*green*). **b**: Chromosomal location, gene structure, and FPKM values in chicken HG and LG lines of linc_gga001850n and its flanking protein-coding gene, *ST3GAL4*. **c**: Chromosomal location, gene structure, and FPKM values in chicken HG and LG lines of linc_gga001994n and its flanking protein-coding gene, *MRC2*. **d**: Two lincRNA genes contain SNPs that have significant associations with special phenotypes

To further investigate the potential functions of lincRNAs, we tested if these transcripts play any role in special biological processes in the NCBI transcriptome projects. To do this, we picked eight diverse conditions from eight projects that encompassed at least four biological replicates (Methods). We retained 68 differentially expressed lincRNAs (*p*-value <0.05 and expression fold change, FC > 2) from six projects using our filtering criteria (Fig. 4a and Additional file 11).

Intriguingly, two differentially expressed lincRNAs, identified from one project (NCBI project ID: SRP028166) where a transcriptome from abdominal fat was analyzed for high-growth (HG) and low-growth (LG) genotype chicken lines at seven weeks of age, revealed potentially important functions. Linc_gga001850n (chr24: 437,952–441,166), a 222 nt transcript encoded by two exons, had a 5.6-fold higher expression in HG than LG abdominal fat chicken lines. The closest protein-coding gene to this transcript, *ST3 beta-galactoside alpha-2,3-sialytransferase 4* (*ST3GAL4*), was also expressed at 2.2 times higher level in HG chickens compared to their LG counterparts **(**Fig. 4a–b **)**. This gene encodes a member of the glycosyltransferase 29 family which is involved in protein glycosylation, and has been shown to be associated with lipid traits in different human ethnic groups [54–56]. Similarly, linc_gga001994n (chr27: 2,426,075–2,428,073) transcribes a 1554 nt lincRNA encoded by two exons and showed a two times higher expression rate in HG compared to LG chicken. Its nearest protein-coding gene, *mannose receptor C type 2* (*MRC2*), had a 2.5 times higher expression level in HG than LG chickens **(**Fig. 4a and c). Previous results have identified *MRC2* as a marker of alternatively activated (M2) macrophages in adipose tissue [57–59]. Weisberg et al. [60] further showed that obesity is associated with macrophage accumulation in adipose tissue. Our analysis suggests that both linc_gga001850n and linc_gga001994n might be associated with adipose tissue development in chicken.

### LincRNA genes containing phenotype associated SNPs

Several studies, particularly GWAS, have investigated the genetic basis of chicken phenotypic traits [19, 20, 26, 61, 62]. These studies have revealed numerous annotated loci in non-coding, especially intergenic, regions that might involve lncRNA genes.

We retrieved 2601 significant SNPs associated with 113 characteristics from 20 previous GWAS [19–38]. Subsequent to location transformation (Methods), this approach yielded 2594 significant SNPs which account for 0.45% of the high-density 600 K array comprising 580,954 SNPs [63]. Our analysis showed that 98 of the significant SNPs are located in 47 of the 4754 lincRNAs (Fig. 4a and Additional file 12). This implies that about 1% of the transcripts we identified might perform important roles in multifarious chicken characteristics including virus resistance, egg or meat production traits, and comb phenotypes. For example, four genome-wide significant SNPs associated with comb phenotypes (i.e., length, height, and weight) are located within linc_gga013241 (chr4: 729,869–741,796), which transcribes two isoforms 3647 bp and 3671 bp in length. In addition, linc_gga000498 (chr1: 65,669,068–65,674,918) also transcribes two isoforms 4923 bp and 1135 bp in length, and probably influences eggshell thickness, weight or strength, because the loci is linked with two other significantly egg-correlated SNPs (Fig. 4a and d).

### LincRNAs under potential artificial selection during chicken domestication

**Fig. 5:**
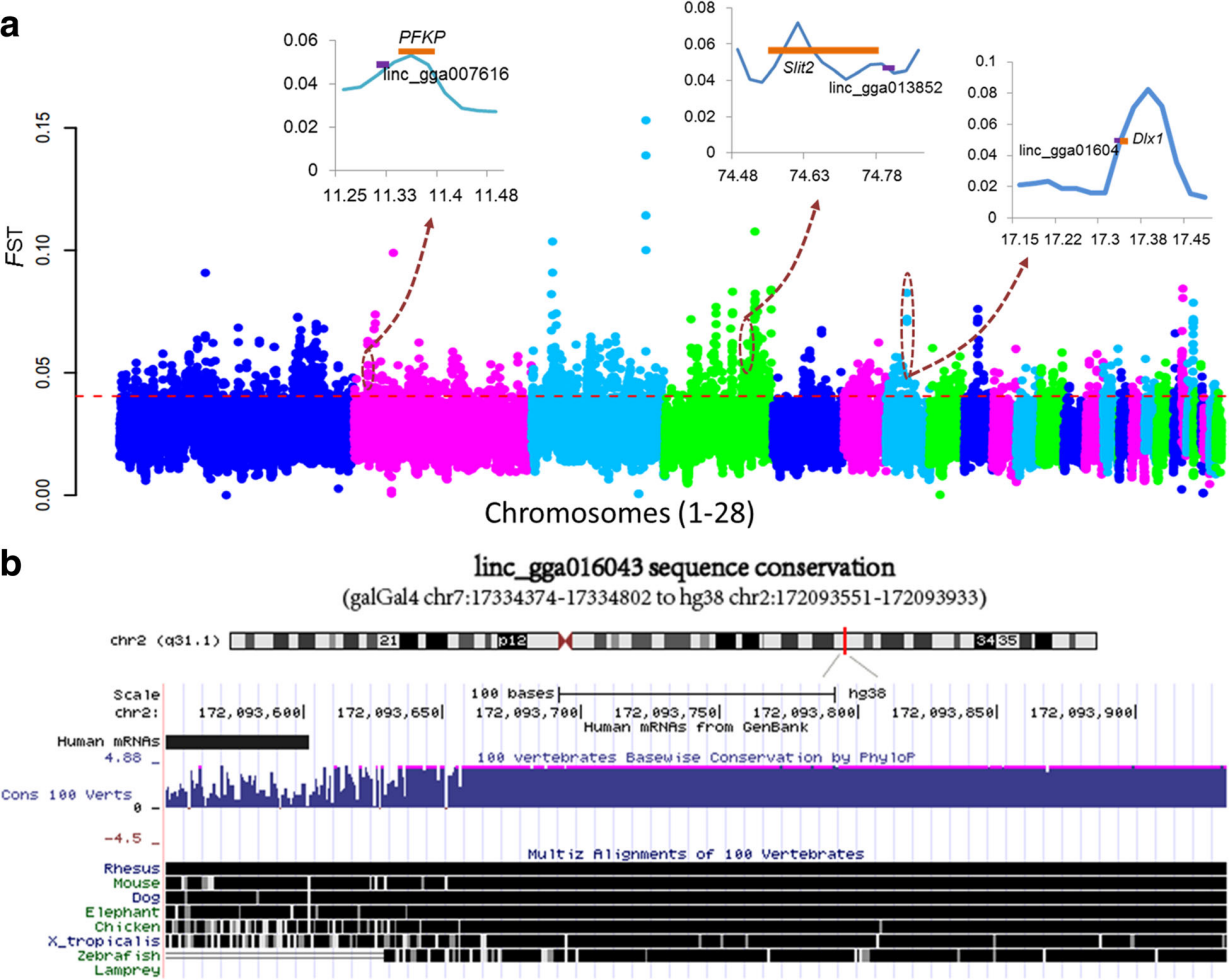
Genome landscape of population differentiation between domestic chicken and RJF. **a**: Manhattan plot for population differentiation evaluated by *F*_ST_. Red dashed line indicates the top 5% rank value of *F*_ST_. Three lincRNAs (linc_gga016043, linc_gga013852, and linc_gga007616) and their flanking protein genes under potential artificial selection are displayed in the top inset images. **b**: Conservation of corresponding human loci of the brain specific lincRNA, linc_gga016043 (from UCSC, 2016)

Numerous phenotypic differences including behavior, morphology, and reproduction, are evident between the domestic chicken and its wild ancestor, RJF. Thus, to further explore potential lincRNA markers of artificial selection during chicken domestication, we evaluated genetic differentiation between domestic chicken (702 genomes) and RJF (36 genomes). We calculated the population differentiation of each SNP between domestic chicken and RJF using fixation index (*F*_ST_) values [64] (Methods) (Fig. 5a). We located a total of 216 lincRNAs genes in the top 5% of *F*_ST_ (Fig. 4a and Additional file 13). Functional annotation was carried out using the tool g:Profiler (version r1622_e84_eg31) [65] for protein-coding genes nearest to these lincRNAs, giving rise to biological process enrichment including development (especially the nervous system), metabolism, and adult behavior (Additional file 14: Figure S4A). We also identified lincRNAs under potential artificial selection by *Pi* and *H*p, realizing 168 and 145 lincRNA genes in the top 5% of *Pi* and *H*p, respectively (Additional file 13 and Additional file 14: Figure S4B). A total of 82 lincRNAs overlapped in both *Pi* and *F*_ST_, 21 in both *H*p and *F*_ST_, and 20 in both *Pi* and *H*p. Thirteen lincRNAs were identified by all of the three methods. Overall, we found 419 potentially selected lincRNAs during chicken domestication.

Interestingly, one brain-specific lincRNA gene, linc_gga016043 (chr7:17,334,374–17,334,802), encodes a 293 nt transcript with two exons, and is substantially conserved within vertebrates (Fig. 5b). Both linc_gga016043 and its proximally located protein-coding gene *Dlx1* are specifically expressed in brain and positively selected (Fig. 5a). *Dlx1* is related to glial cell and neuronal differentiation, as well as apoptosis [66–69]. Indeed, this gene is well-known as a *Distal-less* homeobox transcription factor, functionally redundant to *Dlx2* [67]. Previous work has shown that *Dlx1* is essential for the functional longevity of adult cortical and hippocampal interneurons, as *Dlx1* ^−/−^ mice show a subclass-specific and age-dependent decrease in cortical and hippocampal interneurons caused by apoptosis of *Dlx1*-expressing cells. This decrease leads to a reduction in GABAergic synaptic suppression and results in epilepsy [68]. Another study further found that *Dlx1* and *Dlx2* regulate embryonic forebrain development by balancing neurogenesis and oligodendrogenesis [70]. de Melo et al. [69] demonstrated that these genes are required for the terminal differentiation and survival of retinal ganglion cells in late-born mouse. Thus, it is likely that linc_gga016043 affects the development and function of the nervous system by regulating *Dlx1* expression, and therefore selection of this gene might be linked to chicken behavioral changes after domestication. This shows that lincRNAs probably played an important role in behavior evolution during domestication by influencing brain development.

Moreover, we identified a significant SNP, revealed by GWAS to be associated with egg weight, in a positively selected lincRNA, linc_gga013852 (chr4:74,811,206–74,823,353). This lincRNA has three transcript variants 3068 bp, 2406 bp, and 2083 bp in length created via alternative splicing. Importantly, another seven significant SNPs related to egg weight are within *slit guidance ligand 2* (*Slit2*), the closest protein-coding gene to linc_gga013852. *Slit2* presents strong selection signal (Fig. 5a). It has been reported that the SLIT/ROBO pathway (including the three SLIT ligand genes, *Slit1*, *Slit2,* and *Slit3*, and their receptors *Robo1*, *Robo2*, *Robo3,* and *Robo4*) influences pre-hierarchical follicular development of the hen ovary via intrafollicular autocrine and/or paracrine signaling [71]. Dickinson et al. [72] also showed that this pathway influences luteolysis in female humans.

Our results further show that linc_gga007616 (chr2:11,328,445–11,332,216), a lincRNA specifically expressed in testis, is under selection, and transcribes a 435 nt transcript from two exons. This lincRNA is probably involved in spermatogenesis by regulating the expression of its nearest protein-coding gene, *phosphofructokinase platelet* (*PFKP*), which has selective signals and high expression in testis (Fig. 5a). Indeed, Hering et al. [73] reported the presence of a significant genome-wide SNP marker that lies adjacent to *PFKP*, and is associated with sperm concentration in Holstein-Friesian bulls. It is also known that *PFKP* acts as a marker of oocyte developmental competence in cumulus cells [74], and can indicate whether oocytes are capable of establishing a pregnancy [75]. One key property that supported the long chicken breeding history is egg-laying. Phenotypes of this attribute including egg size, egg number, and laying season, have changed significantly following domestication from RJF. We therefore hypothesize that population differentiation of the two lincRNAs, linc_gga013852 and linc_gga007616, may be associated with the evolution of egg laying in domestic chicken.

Taken together, these results suggest that lincRNAs have played an important role in domestication, where little attention was previously paid.

## Discussion and conclusions

We applied a series of stringent criteria and procedures in this study, and identified more than 4700 candidate lincRNA genes based on 821 transcriptomes. LincRNAs in the chicken genome exhibit similar features to those reported in other species, for instance, a significant expression correlation with adjacent protein-coding genes, and high level of tissue specificity. Enrichment analyses of lincRNA-adjacent protein-coding genes also show that chicken lincRNAs likely regulate transcription, cell proliferation, apoptosis, and development [42, 44–47].

In order to gain deeper insights on the biological significance of lincRNAs, we leveraged data from 48 NCBI projects and curated 68 differentially expressed lincRNA genes (i.e., *p*-value <0.05, FC > 2). These lincRNAs probably influence biological processes such as abdominal fat accumulation in divergent growth genotypes, responses to heat stress in the chicken hepatocellular carcinoma cell line, and sperm mobility in the New Hampshire chicken breed (Additional file 11). Based on these assessments, two lincRNA genes, linc_gga001850n and linc_gga001994n (transcript lengths of 222 nt and 1554 nt, respectively), can be hypothesized to influence abdominal fat accumulation in chicken lines. Indeed, *ST3GAL4*, the nearest protein-coding gene to linc_gga001850n, has been linked with lipid traits in different populations [54–56], while the closest protein-coding gene to linc_gga001994n, *MRC2*, is a marker of M2 macrophages in adipose tissues and is thus related to obesity [57–60]. These two lincRNA genes are therefore promising targets for future studies on chicken breeding and adipose metabolism.

Changes in many phenotypes including behavior, reproduction, and body size, occurred during chicken domestication. Through a whole-genome comparative analysis of RJF and domestic chicken, we identified 419 candidate lincRNAs under selection (Additional file 13). GWAS data and the annotation of neighboring protein-coding genes revealed that lincRNAs probably contributed to chicken domestication by influencing reproductive capability, behavior, and body morphology. For example, the gene linc_gga016043, highly conserved in vertebrates, was likely involved in the evolution of behavior during chicken domestication as it influenced brain development by regulating the expression of its nearest protein-coding gene, *Dlx1*. This protein-coding gene has been shown to be associated with glial cell and neuronal differentiation, as well as apoptosis [66–69]. In addition, linc_gga013852 and linc_gga007616 may modulate chicken fertility by regulating egg laying properties, while their nearest protein-coding genes, *Slit2* and *PFKP*, affect either pre-hierarchical follicular development of hen ovary or bull sperm concentration [71–75]. This study therefore provides evidence for the functional involvement of lincRNAs in chicken domestication.

Overall, our findings show that genetic changes that occurred over the millennia of chicken domestication and development involved not only protein-coding genes, but also lincRNAs. Indeed, it is likely that lincRNAs played a substantial role in this process. Further experimental evidence will provide a deeper understanding of the role of these transcripts in the evolution of complex traits in chicken and other domestic animals.

## Methods

### RNA-sequencing data

A total of 715 RNA-seq data from 48 chicken projects were downloaded from the NCBI website (http://www.ncbi.nlm.nih.gov/). A detailed description of this dataset is presented in Additional file 1. We augmented these data with another set of 195 chicken RNA samples taken from an unpublished study within our research group. Samples were collected from 157 embryo tissues, 28 adult encephalic regions, and ten adults organs, including brain, kidney, heart, eye, and eggs. RNA was isolated using Trizol reagent (Invitrogen) and RNeasy Mini Kits (Qiagen). RNA samples with a RIN value greater than 7.0 were used for library construction and sequencing on the Illumina Hiseq 2000 platform using an insert size of approximately 300 bp.

### Read alignment, transcript assembly, and quantification

Adaptors and low quality reads with insert size shorter than 25 bp or average quality scores less than 18 were trimmed using the software btrim [76]. Sequence fragments were aligned to the chicken genome galGal4.79 (Ensembl v79 [77]) using TopHat2 software (v2.0.14) [78], with defaults except setting ‘--read-mismatches’, ‘--read-edit-dist’, as well as ‘--read-gap-length’ to no more than three bases. Samples with alignment rates (i.e., overall read mapping rates of single-end sequencing libraries and concordant pair alignment rates of paired-end sequencing libraries) greater than or equal to 60% were reserved for subsequent transcript assembly and quantification in Cufflinks software (v2.2.1) [79], using the default parameters and the ‘-GTF-guide’ option. Accepted hits bam files from TopHat2 with the same experimental identification numbers were merged using SAMtools (v0.1.18) [80] into single bam files for subsequent analytical steps. Full command lines are described in Additional file 15.

As described by Zhong et al. [81] and Necsulea et al. [13], strand-specific RNA-seq data can be combined with nonstrand-specific RNA-Seq data ignoring strand information to ensure compatibility between the two types of data. In our assessment, we found that overall read mapping rates, concordant pair alignment rates, as well as gene FPKM values were nearly the same when considering the strandness information or not (Additional file 16: Figure S5). This suggests that treating strand-specific libraries as unstranded would present no significant impact on expression levels in our study. This approach is in agreement with several previous studies [8, 13, 43, 82, 83]. Therefore, for consistency, we set the parameter ‘--library-type’ at default (i.e., fr-unstranded) when running TopHat2 and Cufflinks for all samples. We also excluded mono-exonic transcripts in following analyses, as the presence of canonical (GT-AG) introns in transcript enables prediction of the transcription strand of loci [13].

### Identification of lincRNAs

We used theCuffcompare program in Cufflinks suite to obtain a non-redundant set of transcripts of all Cufflinks processed data. Next, a series of parameters (i.e., exon number, FPKM, and coverage greater than 1, 1, and 3, respectively, and transcript length no less than 200) were used to filter potential false positive transcripts. Newly identified intergenic transcripts were used to detect protein-coding potential using CPC scores [84]. Transcripts with CPC scores less than zero were considered to be potentially non-coding. We used BLASTx [85] to search against a non-redundant protein database to filter potential transcripts. Loci with transcripts that exhibited significant hits (i.e., alignment length greater than 30 bp and e-value less than 0.001) were abandoned, eventually leading to 4754 reliable candidate lincRNAs loci. All candidate lincRNA transcripts were then separately compared with references of known lncRNAs from two lncRNA databases, ALDB (v1.0) [40], and NONCODE (2016) using Cuffcompare (v2.2.1) [79]. Loci with transcript annotated with class codes ‘=’, ‘c’, ‘j’, ‘o’, or ‘p’ were discarded as novel lincRNAs.

### Length and exon number comparisons between lincRNA and protein-coding transcripts

We retrieved transcript information, including chicken protein-coding transcripts as well as lincRNA and protein-coding transcripts in human (GRCh38.p2) and mouse (GRCm38.p3), from the BioMart section of Ensembl 79 [77, 86]. We counted length and exon number of these transcripts, and compared them between lincRNAs and protein-coding genes in the three species respectively.

### Expression correlations between lincRNA and proximal protein-coding genes

Location information of protein-coding genes in the chicken genome was downloaded from the Table Browse of UCSC (University of California, Santa Cruz, UCSC) [87]. The closest protein-coding gene to each lincRNA was then obtained with the ‘closest’ setting in the bedtools software (v2.22.0) [88], using default parameters with the exception of reporting distance with respect to the reference genome (−D = ref).

To examine expression relevance, we calculated PCCs of FPKM values across all samples by paired comparison for three pairs with the same numbers. Specifically, lincRNAs and their corresponding protein-coding genes within 20 kb proximity, neighboring protein-coding gene pairs with distances less than 20 kb, and protein-coding gene pairs selected randomly. Two tests, Student’s *t*- and Wilcoxon, were utilized to check for the significance of expression correlation among the three pairs.

To test whether the 1797 protein-coding genes used for DAVID annotation were expressed in the same tissues as the lincRNAs, we calculated the number of lincRNA/proximal protein-coding gene pairs expressed in a given tissue type. Here, only a lincRNA and its proximal protein-coding gene both with a mean FPKM value larger than 1 [89–91] in one tissue are kept and considered to be expressed in that tissue.

### Calculation of tissue specificity

We calculated the TSI following published formula [48] as follows:

$$ \tau =\frac{\sum_{i=1}^N\left(1-{x}_i\right)}{N-1}. $$

Where, N refers to the number of tissues while x_i_ denotes the expression level in tissue i, and is normalized by the maximal expression value across N tissues.

Because of potential differences in the designs of source studies, we clustered our 821 samples into 21 tissue-related groups via sample coherence and by using the ‘plotClusterTreeSamples’ function in the WGCNA package [49] with the FPKM values of all genes (Fig. 1b and Additional files 2 and 3). Clustering dendrograms comprising samples from markedly different tissues were classed as ‘others’.

We utilized the ‘rsgcc’ package for tissue-specific lincRNA identification, calculating tissue specificity scores as follows:

$$ 1-\min \left(\mathrm{R}(1),\mathrm{R}(2),\dots, \mathrm{R}\left(\mathrm{i}\right),\dots, \mathrm{R}\left(\mathrm{n}\right)\right). $$

Where R(i) = M(i) / E(i); E(i) is the mean or maximal expression value of tissue i, and M(i) is the maximal expression value of other tissues. Thus, a gene is considered to exhibit tissue-specific expression if this score is higher than the parameter ‘tsThreshold’ [51]. We log-transformed FPKM values and applied the maximum value of expression to each tissue-related group. LincRNAs with specificity scores greater than or equal to 0.75 were considered specific to corresponding tissues.

### Functional annotation of lincRNAs using GSEA

We calculated PCCs of expression between each tissue-specific lincRNA and all protein-coding genes for each tissue. Significantly correlated (*p*-value <0.05) protein-coding genes ranked by PCC were chosen for GO enrichment assessment in GSEA [53]. We established an association matrix between tissue-specific lincRNAs and significant GO biological process gene sets (i.e., FDR < 0.25), with the numbers 1, −1, and 0 corresponding to positive, negative, and no significant correlations, respectively. Depending on functional similarity and relevance, GO gene sets were grouped into 11 clusters, and a heatmap plotted for the clustered association matrix (Fig. 3d).

### Differential expression analysis of lincRNAs in eight out of 48 transcriptome projects

Taking into account sample numbers and experimental conditions, we chose eight out of 48 NCBI projects to identify differentially expressed lincRNAs. Detailed information about these projects can be found in NCBI. Briefly, fluctuations of environmental temperature triggers evolutionarily conserved responses in homothermic animals [92]. Projects SRP030116 and SRP038918 characterized transcriptome responses to heat stress in chicken liver and hepatocellular carcinoma cell lines, respectively. In addition, abdominal fat is one of the most important chicken phenotypic and metabolic measurements. Three projects have focused on transcriptional differentiation of abdominal fat in divergently selected chickens; SRP017597 focused on fat and lean chicken lines, while SRP028166 addressed high and low growth genotypes, and SRP058295 looked at modern commercial broiler chickens with high or low feed efficiencies. The bacterium *Campylobacter jejuni* from poorly cooked chicken meat often causes human bacterial gastroenteritis. Thus, to investigate the resistance mechanisms of birds to *C. jejuni* colonization, RNA-seq analysis of whole caecum from *C. jejuni*-susceptible and resistant chickens was carried out in project SRP018692 [93]. Project SRP042038 analyzed expression differences in whole testes between high and low sperm mobility lines of New Hampshire breed chickens. Finally, meat production in domesticated chicken has been under intensive selective pressure. Davis et al. [94] identified differentially enriched genes in the post-hatching pectoralis major muscle between modern and legacy broiler lines (project SRP052755).

We detected differential expression of lincRNAs under the variable conditions seen in the eight projects discussed above as previously described [95, 96]. In detail, we performed Student’s *t*-test for log2 (FPKM +1), and assessed statistically significant differences using a Benjamini-Hochberg-corrected *p*-value <0.05. Significant lincRNAs with an FC greater than 2 or less than 0.5, as well as a mean FPKM from at least one of two contrary conditions larger than 1 were considered differentially expressed. By using these filtering criteria, we found 68 differentially expressed lincRNAs in six projects. No differentially expressed lincRNAs were found in the other two projects.

### Phenotype associated SNPs

We collected a total of 2601 genome-wide significant SNPs, involving about 113 features from 20 previous GWAS studies, ignoring association test models performed in these studies. In the case of SNPs based on galGal3 assembly, we utilized LiftOver tool of web version (http://genome.ucsc.edu/cgi-bin/hgLiftOver) to align them with galGal4. Because SNPs based on galGal3 were identified using Illumina 60 K Chicken SNP Beadchip with 52,303 SNPs [97], which forms part of the Affymetrix® Axiom® array 600 K Array consisting of 580,954 SNPs [63], we used the 600 K array for further ratio calculations.

### Population differentiation of lincRNA genes between domestic chicken and RJF

Whole genomes from 36 RJFs and 702 domestic chickens were obtained from an unpublished project within our laboratory, and taken through an analytical pipeline used by previous studies [2, 98, 99]. In detail, we trimmed off adaptors and low-quality reads with insert size shorter than 25 bp or average quality scores less than 20 using btrim [76], and then used the BWA-MEM algorithm [100] of the BWA software package (v0.7.12-r1039) for genomic sequence alignment with options ‘-t 12 -M -R’. Aligned bam files were then sorted and duplicates marked using the Picards package (v1.56). SNP calls were carried out via Genome Analysis Toolkit (GenomeAnalysisTK-2.6-4, GATK) [101]. Population differentiation between RJF and domestic chicken was evaluated using *F*_ST_ for each SNP as previously described [64]. A 50 kb sliding window size was used for the *F*_ST_ statistic, and regions in the top 5% of results were regarded as potential candidates for artificial selection. We also identified the lincRNAs potentially under artificial selection by *Pi* (nucleotide diversity) and *H*_p_ (heterozygosity). Nucleotide diversities (Δπ or ΔPi) = πRJF − πVC were also calculated using a sliding window analysis with a window size of 50 kb and a step size of 25 kb as described elsewhere [98]. *H*_p_ was calculated following published formula [1] in sliding 40-kb windows: *H*p = 2ΣnMAJΣnMIN/(ΣnMAJ + ΣnMIN)^2^, where nMAJ and nMIN represent the most and least abundant allele respectively, while ΣnMAJ and ΣnMIN are the sums of nMAJ and nMIN, respectively, for all SNPs in the window.

## Additional files

Additional file 1: Sample information of 821 transcriptomes. (XLSX 83 kb)

Additional file 2: Samples belonging to the 21 WGCNA cluster groups. (XLSX 48 kb)

Additional file 3: The expression cluster tree from WGCNA. (PDF 760 kb)

Additional file 4: Figure S1. LincRNAs’ comparisons to other databases and fundamental features. **A:** Overlaps between each two of our putative lincRNAs (black), NONCODE lncRNAs (purple) and ALDB lincRNAs (cyan). Digits in square brackets show total lncRNA or lincRNA genes. **B:** Expression (normalized by log2 (FPKM + 1)) of lincRNA (orange) and protein-coding genes (green). **C:** Length of lincRNA transcripts. Red dashed represents the median length for all transcripts. **D:** Exon number of lincRNA transcripts, with mean marked by red dashed. (TIFF 150 kb)

Additional file 5: Length and exon number comparisons of lincRNA and protein-coding transcripts. (DOC 32 kb)

Additional file 6: Figure S2. Length and exon number comparisons between lincRNA and protein-coding transcripts in human and mouse. **A:** Length and exon number of lincRNA transcripts in human. **B:** Length and exon number of protein-coding transcripts in human. **C:** Length and exon number of lincRNA transcripts in mouse. **D:** Length and exon number of protein-coding transcripts in mouse. Red dashed lines represent the median length and average of exon number, respectively. (TIFF 188 kb)

Additional file 7: Number and parts of DAVID annotation terms of proximal protein-coding genes within 20 kb proximity to lincRNAs expressed in the 20 tissue groups. (DOC 48 kb)

Additional file 8: DAVID annotation of proximal protein-coding genes within 20 kb proximity to lincRNAs expressed in the 20 tissue groups. (XLSX 114 kb)

Additional file 9: Figure S3. Characteristics of tissue specificity of lincRNAs and protein coding genes. **A-C:** Comparisons of TSI among lincRNAs, their flanking protein-coding genes and other protein-coding genes; **A)** for all lincRNAs; **B)** for lincRNAs with TSI large than 0.95; **C)** for tissue specific lincRNAs calculated from “rsgcc”. Wilcoxon test *p*-values were showed on the top. **D:** Expression heatmap of tissue-specific lincRNAs identified using TSI. Columns represent samples while rows represent lincRNAs. (TIFF 279 kb)

Additional file 10: Tissue-specific lincRNAs identified by TSI and “rsgcc”. (XLSX 222 kb)

Additional file 11: Differentially expressed lincRNAs from six NCBI projects. (XLSX 15 kb)

Additional file 12: Significant trait-correlated SNPs retrieved from 20 studies based on GWAS, and lincRNAs with significant SNPs. (XLSX 274 kb)

Additional file 13: LincRNAs located in the top 5% of *F*_ST_, *Pi* and *H*_p_ statistics. (XLSX 46 kb)

Additional file 14: Figure S4. LincRNAs under potential artificial selection. **A:** Significant categories enriched among protein-coding genes adjacent to lincRNAs that were located in the top 5% of *F*_ST_. **B:** LincRNAs potentially under artificial selection identified by *F*_ST_, *Pi* and *H*_p_. (TIFF 168 kb)

Additional file 15: Full command lines of our major computational pipelines. (DOC 37 kb)

Additional File 16: Figure S5. Strandedness’ impact on expression estimation of strand-specific libraries. **A:** Comparisons of overall read mapping rates and the concordant pair alignment rates between ‘fr-firststrand’ and ‘fr-unstranded’ settings for the 124 firststrand-specific samples. **B:** PCCs of gene FPKM values between ‘fr-firststrand’ and ‘fr-unstranded’ settings for the 124 firststrand-specific samples. (TIFF 756 kb)

Additional file 17: Detailed feature information of the 4754 lincRNA genes. (GTF 3947 kb)

Additional file 18: The information of proximal protein-coding genes to lincRNAs. (XLSX 436 kb)

## Abbreviations

bp: Base pairs
FPKM: Fragments Per Kilobase of exon model per Million mapped fragments
*F*_ST_: Fixation index
GO: Gene ontology
GWAS: Genome-wide association studies
HG: High-growth
kb: Kilobases
LG: Low-growth
lincRNAs: Long intergenic noncoding RNAs
lncRNAs: Long noncoding RNAs
NCBI: The US National Center for Biotechnology Information
nt: Nucleotides
PCC: Pearson correlation coefficients
RJFs: Red junglefowls
SNPs: Single-nucleotide polymorphisms
TSI: Tissue specificity index
