# Supplementary figures and images for: Annotating long intergenic non-coding RNAs under artificial selection during chicken domestication
Source: BMC Evol Biol. 2017 Aug 15;17:192. doi: 10.1186/s12862-017-1036-6 (PMC5558714; doi:10.1186/s12862-017-1036-6)

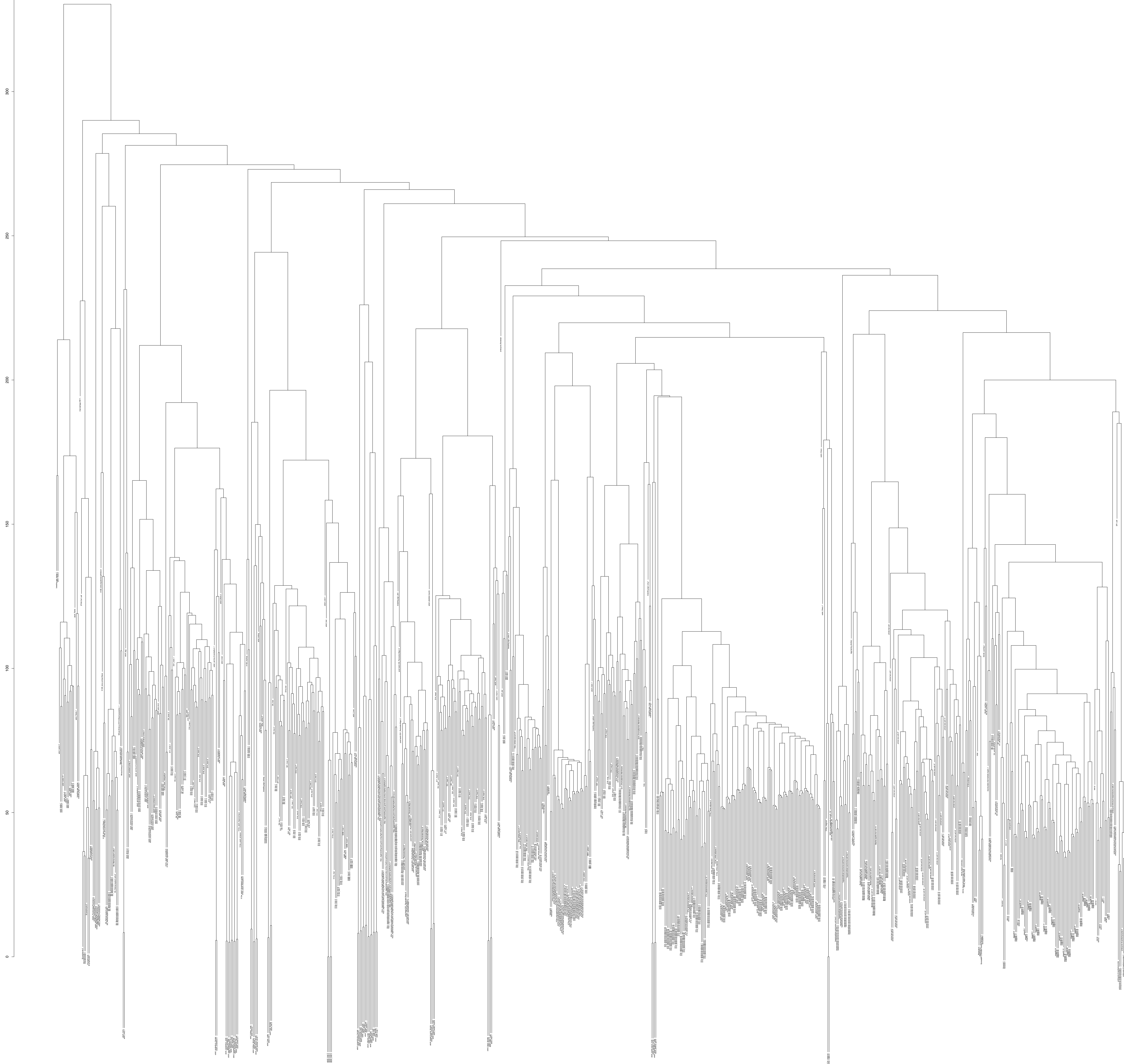

Supplement: Supplementary file 3 — The expression cluster tree from WGCNA. (PDF 760 kb) [file 12862_2017_1036_MOESM3_ESM.pdf]

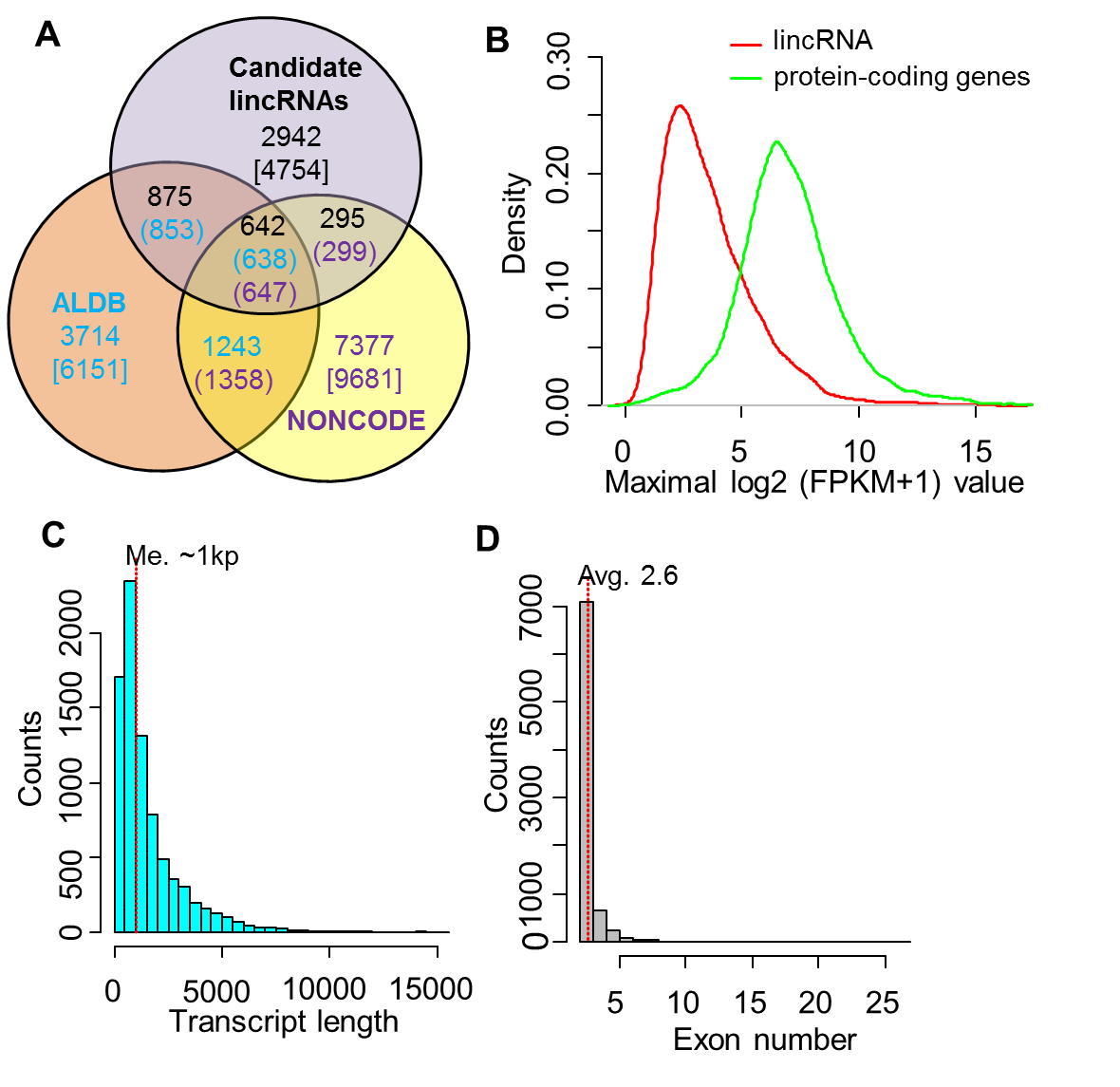

Supplement: Supplementary file 4 — LincRNAs’ comparisons to other databases and fundamental features. A: Overlaps between each two of our putative lincRNAs (black), NONCODE lncRNAs (purple) and ALDB lincRNAs (cyan). Digits in square brackets show total lncRNA or lincRNA genes. B: Expression (normalized by log2 (FPKM + 1)) of lincRNA (orange) and protein-coding genes (green). C: Length of lincRNA transcripts. Red dashed represents the median length for all transcripts. D: Exon number of lincRNA transcripts, with mean marked by red dashed. (TIFF 150 kb) [file 12862_2017_1036_MOESM4_ESM.tif]

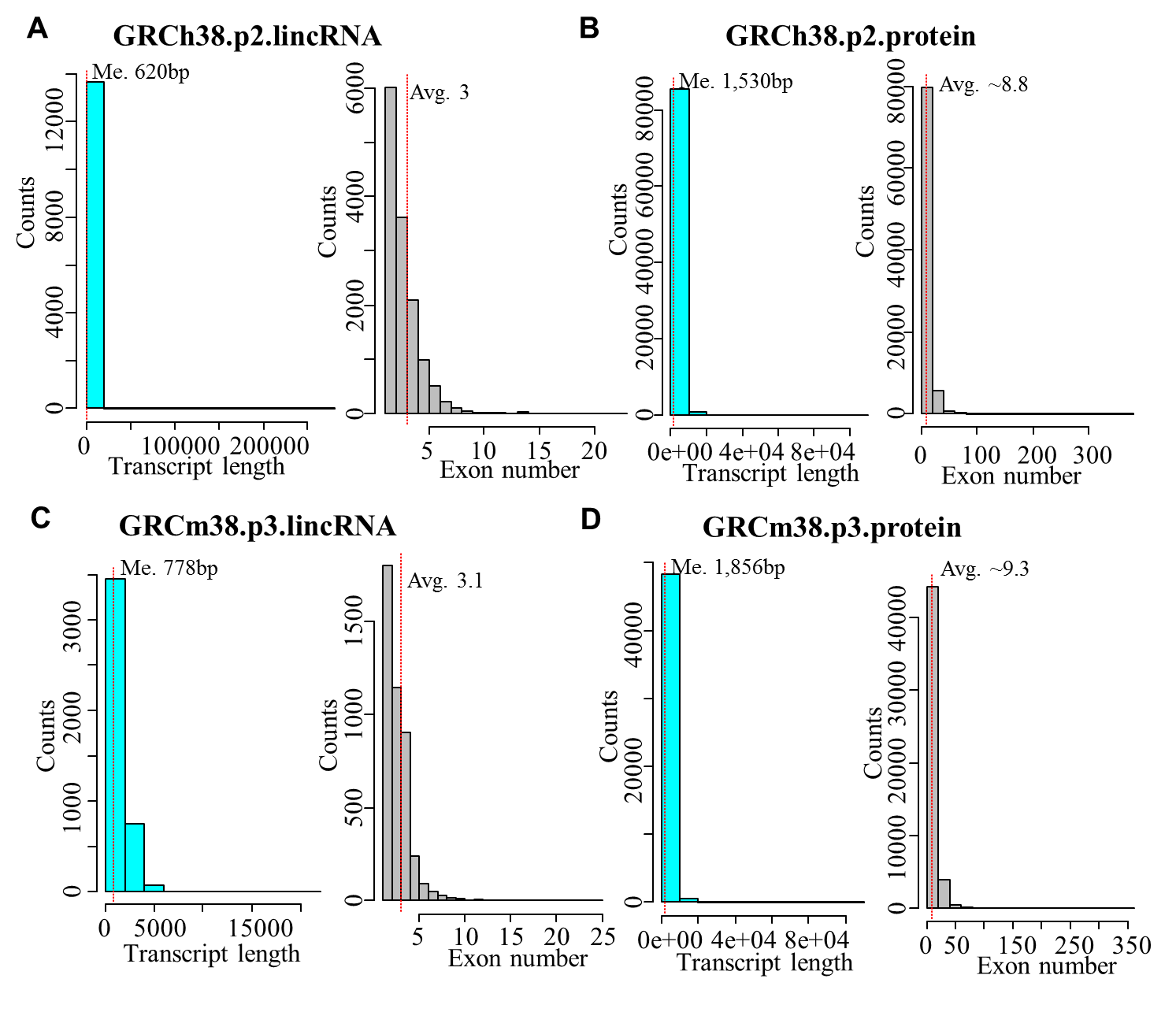

Supplement: Supplementary file 6 — Length and exon number comparisons between lincRNA and protein-coding transcripts in human and mouse. A: Length and exon number of lincRNA transcripts in human. B: Length and exon number of protein-coding transcripts in human. C: Length and exon number of lincRNA transcripts in mouse. D: Length and exon number of protein-coding transcripts in mouse. Red dashed lines represent the median length and average of exon number, respectively. (TIFF 188 kb) [file 12862_2017_1036_MOESM6_ESM.tif]

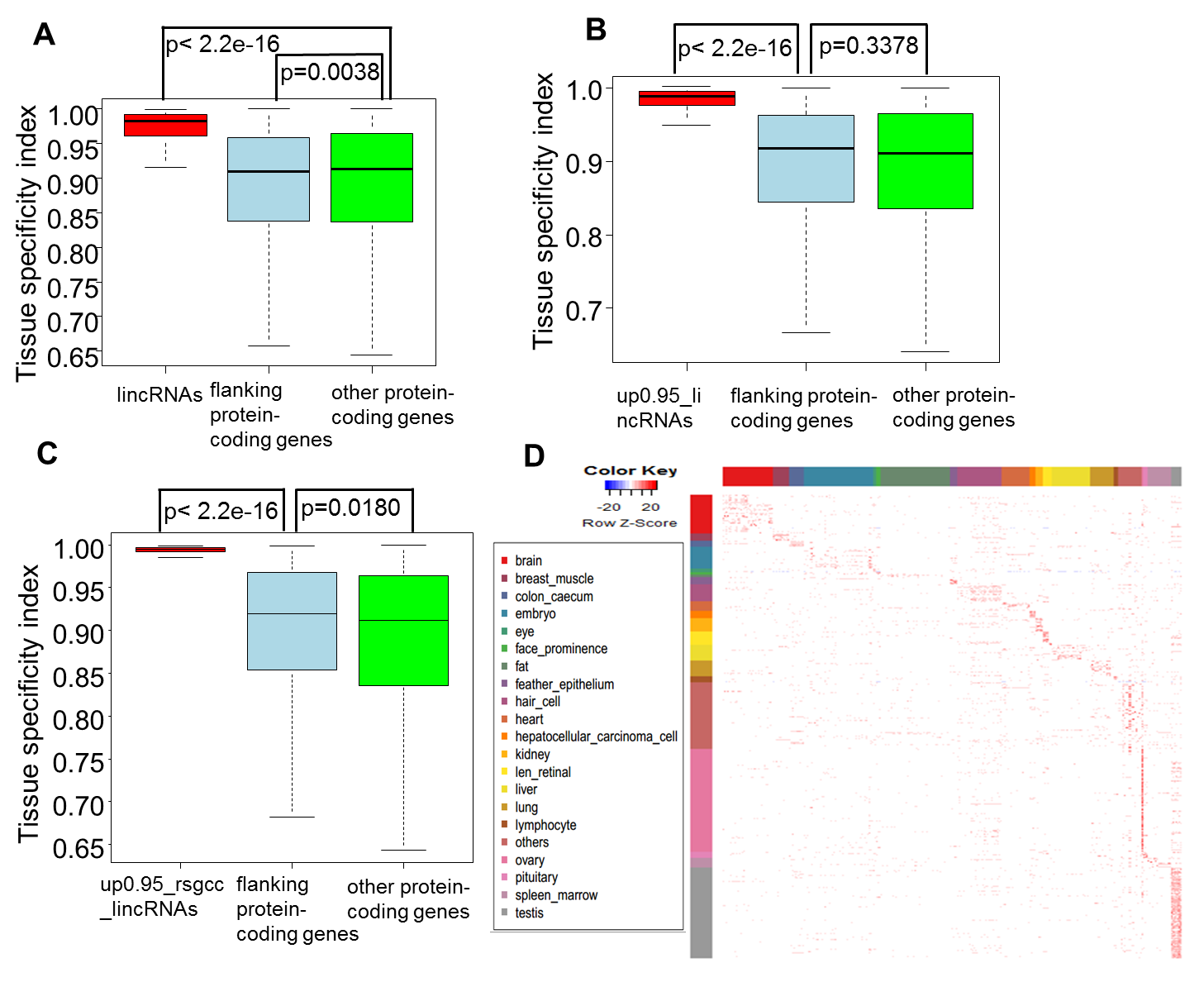

Supplement: Supplementary file 9 — Characteristics of tissue specificity of lincRNAs and protein coding genes. A-C: Comparisons of TSI among lincRNAs, their flanking protein-coding genes and other protein-coding genes; A) for all lincRNAs; B) for lincRNAs with TSI large than 0.95; C) for tissue specific lincRNAs calculated from “rsgcc”. Wilcoxon test p-values were showed on the top. D: Expression heatmap of tissue-specific lincRNAs identified using TSI. Columns represent samples while rows represent lincRNAs. (TIFF 279 kb) [file 12862_2017_1036_MOESM9_ESM.tif]

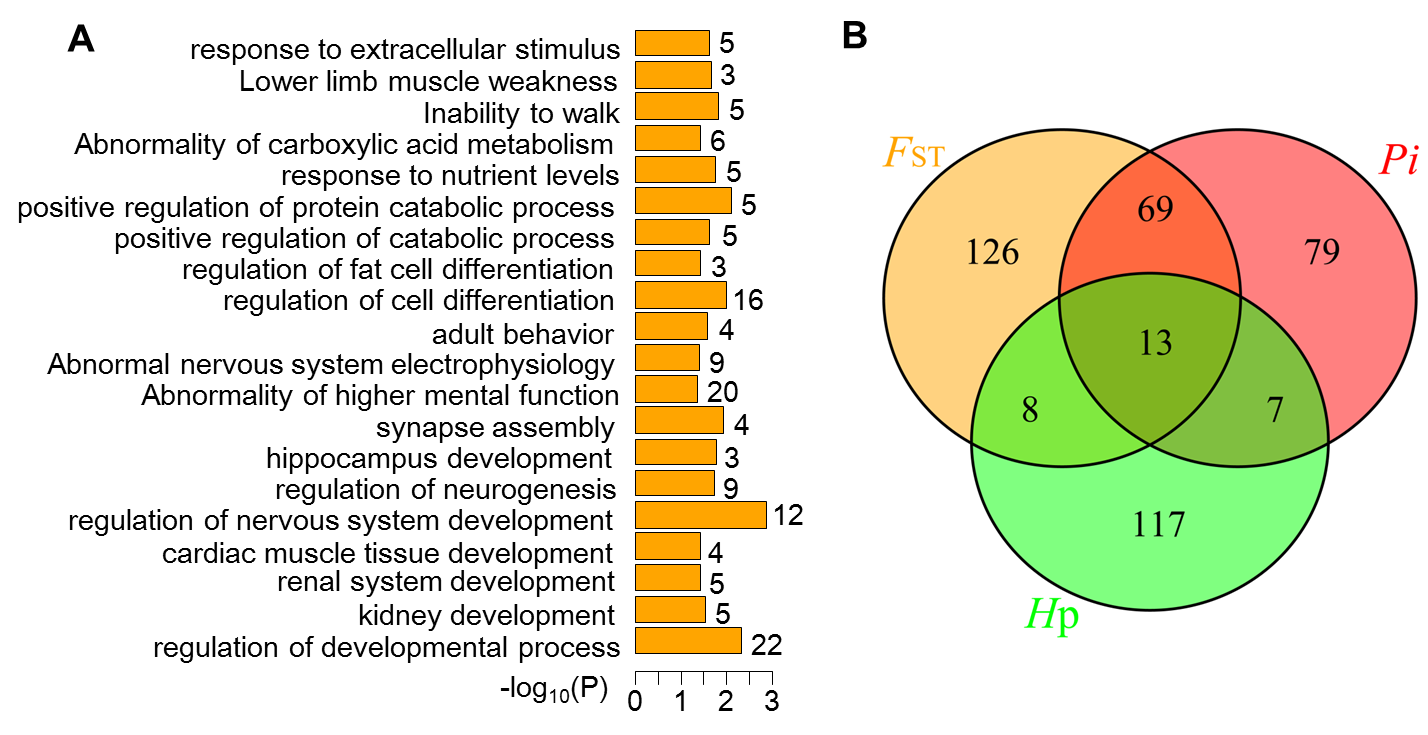

Supplement: Supplementary file 14 — LincRNAs under potential artificial selection. A: Significant categories enriched among protein-coding genes adjacent to lincRNAs that were located in the top 5% of F ST. B: LincRNAs potentially under artificial selection identified by F ST, Pi and H p. (TIFF 168 kb) [file 12862_2017_1036_MOESM14_ESM.tif]

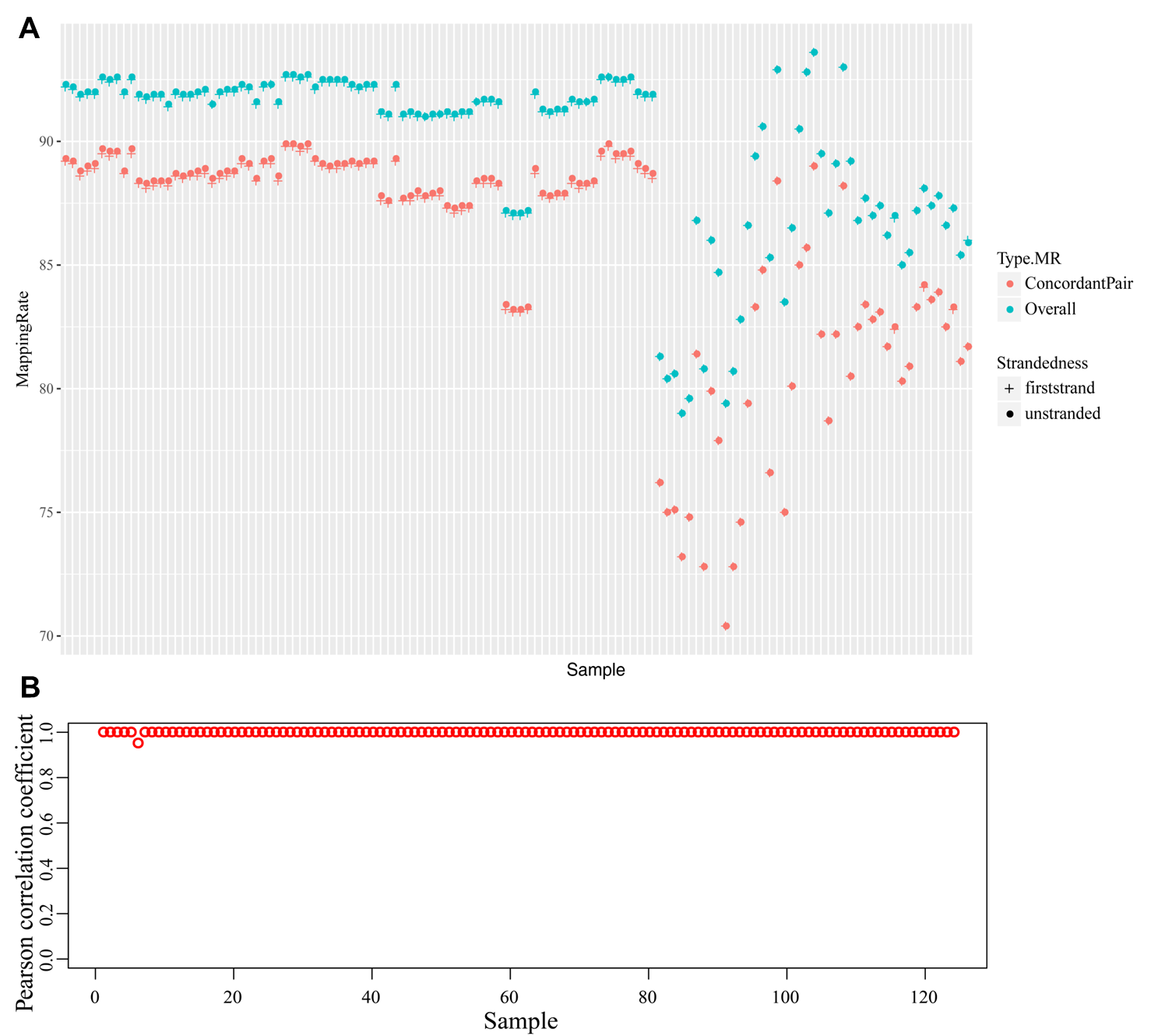

Supplement: Supplementary file 16 — Strandedness’ impact on expression estimation of strand-specific libraries. A: Comparisons of overall read mapping rates and the concordant pair alignment rates between ‘fr-firststrand’ and ‘fr-unstranded’ settings for the 124 firststrand-specific samples. B: PCCs of gene FPKM values between ‘fr-firststrand’ and ‘fr-unstranded’ settings for the 124 firststrand-specific samples. (TIFF 756 kb) [file 12862_2017_1036_MOESM16_ESM.tif]
