# Supplementary material for: Annotating long intergenic non-coding RNAs under artificial selection during chicken domestication
Source: BMC Evol Biol. 2017 Aug 15;17:192. doi: 10.1186/s12862-017-1036-6 (PMC5558714; doi:10.1186/s12862-017-1036-6)
Supplement: Supplementary file 5 — Length and exon number comparisons of lincRNA and protein-coding transcripts. (DOC 32 kb) [file 12862_2017_1036_MOESM5_ESM.doc]

| Species | category | Gene number | Transcript number | Transcript length range (bp) | Transcript length median (bp) | Transcript length difference between lincRNAs/protein-coding genes (bp) | Exon number range | Exon number average | Exon number difference between lincRNAs/protein-coding genes |
| --- | --- | --- | --- | --- | --- | --- | --- | --- | --- |
| Galgal4 | lincRNAs | 4754 | 8134 | 200 - 15386 | **1004** | -2080 | 2 - 27 | **2.6** | -8.3 |
|  | protein-coding genes | 15508 | 16354 | 108 - 92850 | **3084** |  | 1 - 269 | **10.9** |  |
| GRCh38.p2 | lincRNAs | 7839 | 13652 | 61 - 267372 | **620** | -910 | 1 - 23 | **3.0** | -5.8 |
|  | protein-coding genes | 21810 | 86376 | 956 - 109224 | **1530** |  | 1 - 363 | **8.8** |  |
| GRCm38.p3 | lincRNAs | 3006 | 4291 | 88 - 20771 | **778** | -1078 | 1 - 25 | **3.1** | -6.2 |
|  | protein-coding genes | 22154 | 48717 | 24 - 106824 | **1856** |  | 1 - 347 | **9.3** |  |

**Length and exon number comparisons of lincRNA and protein-coding transcripts**
