# Supplementary material for: Annotating long intergenic non-coding RNAs under artificial selection during chicken domestication
Source: BMC Evol Biol. 2017 Aug 15;17:192. doi: 10.1186/s12862-017-1036-6 (PMC5558714; doi:10.1186/s12862-017-1036-6)
Supplement: Supplementary file 7 — Number and parts of DAVID annotation terms of proximal protein-coding genes within 20 kb proximity to lincRNAs expressed in the 20 tissue groups. (DOC 48 kb) [file 12862_2017_1036_MOESM7_ESM.doc]

**Number and parts of DAVID annotation terms of proximal protein-coding genes within 20 kb proximity to lincRNAs in the 20 tissue groups**

| Tissue type | Number of expressed protein-coding genes | Number of expressed lincRNAs | Part of DAVID annotation terms  (Pvalue or EASE Score) |
| --- | --- | --- | --- |
| brain | 308 | 316 | GO:0042177~negative regulation of protein catabolic process (0.007811);  GO:0045471~response to ethanol (0.019476);  Differentiation (0.02139); |
| breast_muscle | 160 | 165 | GO:0042177~negative regulation of protein catabolic process (0.001341);  GO:0001077~transcriptional activator activity, RNA polymerase II core promoter proximal region sequence-specific binding (0.004658)  GO:0002062~chondrocyte differentiation (0.039641) |
| colon_caecum | 293 | 305 | GO:0016477~cell migration (0.001194);  GO:0042127~regulation of cell proliferation (0.009743);  GO:0004672~protein kinase activity (0.011429) |
| embryo | 216 | 223 | Translation regulation (0.003088);  GO:0060348~bone development (0.006079);  GO:0045600~positive regulation of fat cell differentiation (0.011994) |
| eye | 232 | 239 | GO:0060348~bone development (0.007106);  GO:0043588~skin development (0.007106);  GO:2001200~positive regulation of dendritic cell differentiation (0.036138) |
| face_prominence | 261 | 273 | GO:0007569~cell aging (0.003148);  GO:0060348~bone development (0.009599);  GO:0045600~positive regulation of fat cell differentiation (0.018684) |
| fat | 166 | 171 | GO:0005925~focal adhesion (0.010754);  GO:0007219~Notch signaling pathway (0.019816);  GO:0045600~positive regulation of fat cell differentiation (0.047836) |
| feather_epithelium | 163 | 166 | GO:0009888~tissue development (0.016458);  GO:0071800~podosome assembly (0.036899);  response to UV-A (0.048896) |
| hair_cell | 346 | 363 | GO:0010634~positive regulation of epithelial cell migration (0.006732);  GO:0009617~response to bacterium (0.022557);  GO:0032924~activin receptor signaling pathway (0.01196) |
| heart | 194 | 200 | GO:0007507~heart development (6.25E-04);  GO:0060045~positive regulation of cardiac muscle cell proliferation (0.011097);  GO:0007219~Notch signaling pathway (0.03082) |
| hepatocellular_carcinoma_cell | 251 | 260 | GO:0097421~liver regeneration (0.002176);  Transcription regulation (0.008198);  IPR008964:Invasin/intimin cell-adhesion (0.03639) |
| kidney | 280 | 287 | GO:0007219~Notch signaling pathway (0.003529);  GO:0003094~glomerular filtration (0.007094);  gga04320:Dorso-ventral axis formation (0.008464) |
| len_retinal | 290 | 299 | GO:0035278~miRNA mediated inhibition of translation (0.001031);  GO:0030534~adult behavior (0.006071);  GO:0023014~signal transduction by protein phosphorylation (0.03234) |
| liver | 163 | 168 | gga01100:Metabolic pathways (0.023961);  GO:0007219~Notch signaling pathway (0.024208);  GO:0009408~response to heat (0.041473) |
| lung_CM | 244 | 251 | GO:0003094~glomerular filtration (0.005484);  IPR001723:Steroid hormone receptor (0.033103); |
| lymphocyte | 153 | 157 | Translation regulation (0.018197);  Prenylation (0.024061);  GO:0009408~response to heat (0.034915) |
| ovary | 607 | 645 | Transcription regulation (7.65E-05);  GO:0003007~heart morphogenesis (5.94E-04);  GO:0048565~digestive tract development (0.0047853);  GO:0018108~peptidyl-tyrosine phosphorylation (0.0069982);  GO:0007411~axon guidance (0.019125);  GO:0001764~neuron migration (0.0222937);  GO:0042416~dopamine biosynthetic process (0.0223926);  GO:0071376~cellular response to corticotropin-releasing hormone stimulus (0.0223926);  GO:0007399~nervous system development (0.0224214);  GO:0030335~positive regulation of cell migration (0.0236726) |
| pituitary | 333 | 343 | GO:0023014~signal transduction by protein phosphorylation (0.037466);  GO:0061630~ubiquitin protein ligase activity (0.038113) |
| spleen_marrow | 219 | 223 | GO:0004672~protein kinase activity (0.014738);  GO:0001649~osteoblast differentiation (0.015909) |
| testis | 587 | 623 | GO:0005925~focal adhesion (6.80E-04);  gga05168:Herpes simplex infection (0.011103);  gga04114:Oocyte meiosis (0.036132) |
| total | 5,466 | 5,677 | - |
| unique total | 1,333 | 1,481 | - |
