# Supplementary material for: Annotating long intergenic non-coding RNAs under artificial selection during chicken domestication
Source: BMC Evol Biol. 2017 Aug 15;17:192. doi: 10.1186/s12862-017-1036-6 (PMC5558714; doi:10.1186/s12862-017-1036-6)
Supplement: Supplementary file 15 — Full command lines of our major computational pipelines. (DOC 37 kb) [file 12862_2017_1036_MOESM15_ESM.doc]

**Full command lines of the major computational pipelines**

**a.** Adaptors and Reads trim by btrim

For single-end reads:

btrim64 -S -q -s <summary file> -l 25 -a 18 -o <output file> -t <sequence file>

For paired-end reads, independently do the above step for each end reads, and run the following script to re-synchronize the two end reads:

perl paired_end_trim.pl <summary file from left reads> <summary file from right reads > <output file for left reads from the first step> <output file for right reads from the first step>

**b.** tophat2 for reads mapping

tophat2 -p 6 -N 3 --read-gap-length 3 --read-edit-dist 3 -G <referenc.gtf> -o <output file> <bowtie_index of referenc.fa> < btrim.reads1.pe> < btrim.reads2.pe>

**c.** Cufflinks suite for transcript assembling and expression evolution

cufflinks -p 2 -g < referenc.gtf > -o <output file> accepted_hits.bam

cuffcompare -r <sRNA.removed.reference.gtf> -R -C -V -s <referenc.fa> -o <output file> -i < transcripts_gtf_list>

cufflinks -p 2 -G <filtered gtf file from cuffcompare> -o <output file> <accepted_hits.bam from TopHat2>

**d.** CPC for calculating protein-coding potential scores

cpc-0.9-r2/bin/run_predict.sh <input file> <output file> <working directory> <output_evidence_plot_feat_base>

**e.** blastx to search against a non-redundant protein database to filter potential transcripts

blastx -query <input file> -db 20160404nr -num_threads 6 -evalue 0.001 -max_target_seqs 1 -outfmt 6 -out <output file>

**f.** Our population genomic analysis follows the pipeline same to the previous works ^[1-3]^.

References

1. Wang M-S, Zhang R-w, Su L-Y, Li Y, Peng M-S, Liu H-Q, Zeng L, Irwin DM, Du J-L, Yao Y-G et al: Positive selection rather than relaxation of functional constraint drives the evolution of vision during chicken domestication. Cell Res. 2016; 26(5):556-573.

2. Wang M-S, Huo Y-X, Li Y, Otecko NO, Su L-Y, Xu H-B, Wu S-F, Peng M-S, Liu H-Q, Zeng L et al: Comparative population genomics reveals genetic basis underlying body size of domestic chickens. Journal of Molecular Cell Biology. 2016; 8(6):542-552.

3. Wang M-S, Li Y, Peng M-S, Zhong L, Wang Z-J, Li Q-Y, Tu X-L, Dong Y, Zhu C-L, Wang L et al: Genomic analyses reveal potential independent adaptation to high altitude in Tibetan chickens. Molecular Biology and Evolution. 2015.
